# Bone Cement Implantation Syndrome Causing Intraoperative Cardiac Arrest in a Nonagenarian: A Case Report

**DOI:** 10.7759/cureus.93622

**Published:** 2025-09-30

**Authors:** Binyam M Habte, Yoseph M Habte, Makida M Habte, Esimael M Abdu, Selamawit G Jima

**Affiliations:** 1 Department of Medicine, University of Gondar, Gondar, ETH; 2 Department of Medicine, Ethio Tebib Hospital, Addis Ababa, ETH; 3 Department of Medicine, Bethel Medical College, Addis Ababa, ETH; 4 Department of Surgery, Teklehaimanot General Hospital, Addis Ababa, ETH

**Keywords:** bone cement implantation syndrome, cardiac arrest, case report, cemented hip hemiarthroplasty, polymethylmethacrylate (pmma)

## Abstract

Bone cement implantation syndrome (BCIS) is a rare but potentially fatal complication of cemented orthopedic procedures, most commonly hip hemiarthroplasty in elderly patients. It is characterized by acute cardiovascular and respiratory compromise that can progress to cardiac arrest. We present the case of a 90-year-old woman with long-standing hypertension who sustained a left intertrochanteric femoral fracture following a fall. She was scheduled for cemented hemiarthroplasty and underwent spinal anesthesia. Approximately five minutes after insertion of polymethylmethacrylate bone cement, she developed profound hypoxia, severe hypotension, and bradycardia, which rapidly progressed to cardiac arrest. Cardiopulmonary resuscitation (CPR) was initiated immediately, including intubation, mechanical ventilation with 100% oxygen, intravenous adrenaline, and advanced life support measures. Return of spontaneous circulation was achieved after four cycles of high-quality CPR, and the procedure was completed successfully. Postoperatively, she required intensive care with vasopressor support and mechanical ventilation but was gradually stabilized, extubated, and discharged home in good condition. This case underscores the life-threatening potential of BCIS, the importance of early recognition, and the critical role of timely multidisciplinary resuscitative interventions in determining survival and functional recovery.

## Introduction

Bone cement implantation syndrome (BCIS) is a rare but potentially fatal complication associated with the use of polymethylmethacrylate (PMMA) bone cement during orthopedic procedures, particularly cemented hip arthroplasty [1]. Although the exact incidence is difficult to determine due to variable definitions and underreporting, BCIS grade 3 occurs in approximately 1.7% of cemented hip hemiarthroplasties and is associated with substantial perioperative morbidity and mortality. BCIS grade 3, the most severe form, carries a particularly poor prognosis, with a 30-day mortality rate of 88% among patients undergoing cemented hip hemiarthroplasty for femoral neck fracture [2]. The syndrome is characterized by a spectrum of clinical manifestations, ranging from mild hypoxia or hypotension to profound cardiovascular collapse and cardiac arrest [1,3]. These events typically occur intraoperatively at critical moments such as cement pressurization, prosthesis insertion, or joint reduction, though milder presentations have also been described in the immediate postoperative period [1].

Here, we present the case of a 90-year-old woman who developed severe intraoperative BCIS with cardiac arrest during cemented hemiarthroplasty for an intertrochanteric fracture. The case highlights the importance of vigilance, early recognition, and multidisciplinary management in mitigating the adverse outcomes of this life-threatening complication.

## Case presentation

**Figure 1:**
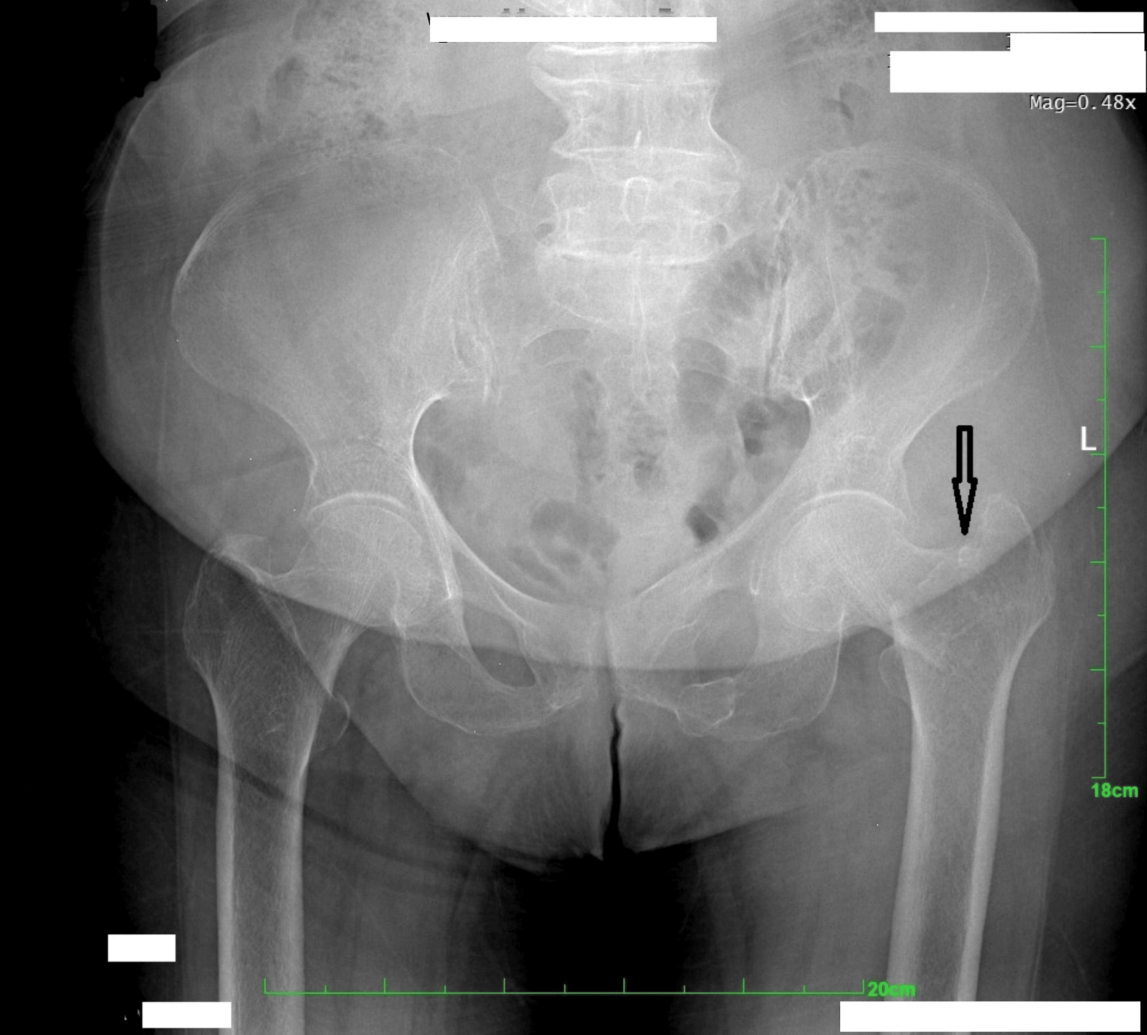
Pelvic radiograph demonstrating a left intertrochanteric femoral fracture

A 90-year-old woman with a 30-year history of hypertension, well controlled with amlodipine 5 mg orally once daily, presented to the emergency department three hours after slipping and falling from her bed while attempting to go to the toilet. She reported severe pain and an inability to move her left lower extremity. Pelvic radiographs confirmed a left femoral intertrochanteric fracture (Figure 1).

She was initiated on prophylactic unfractionated heparin for venous thromboembolism prevention and scheduled for hemiarthroplasty the following day. On preoperative assessment, the patient was fully conscious, alert, and cooperative. Her blood pressure was 140/70 mmHg, her pulse rate was 76 beats per minute, and other systemic examinations were unremarkable. Laboratory investigations, including complete blood count and organ function tests, were within normal limits (Table 1). Echocardiography revealed mild aortic regurgitation, while the electrocardiogram was normal.

**Table 1:** Laboratory investigations with corresponding results and reference values All laboratory investigations were within normal limits, except for postoperative anemia, for which the patient received a transfusion of packed red blood cells. Additionally, a transient positive fecal occult blood test was observed following NSAID administration, which resolved after discontinuation of NSAIDs and adjustment of anti-pain medications. HIV: human immunodeficiency virus, NSAID: non-steroidal anti-inflammatory drug

Laboratory parameters	Day 1	Day 7	Discharge	Normal value
Complete blood count				
White blood cell	9.8 × 10^3^/µL	10.8 × 10^3^/µL	11.0 × 10^3^/µL	4.0-11.0 × 10^3^/µL
Hemoglobin	14.0 g/dL	9 g/dL	12.8 g/dL	13.5-17.5 g/dL
Platelet	422 × 10^3^/µL	400 × 10^3^/µL	348 × 10^3^/µL	150-450 × 10^3^/µL
Lymphocyte percentage	23.00%	20%	19%	15-50%
Neutrophil percentage	68.10%	76%	78%	45-80%
Metabolic panel				
Creatinine	0.94 mg/dl	0.86 mg/dl	0.91 mg/dl	0.67-1.17 mg/dl
Urea	29 mg/dl	26 mg/dl	27 mg/dl	43 mg/dl
Na+	136 mmol/l	139 mmol/l	142 mmol/l	136-145 mmol/l
K+	4.4 mmol/l	3.9 mmol/l	4.1 mmol/l	3.5-5.1 mmol/l
Albumin	4.6 g/dL	3.9 g/dl	4.2 g/dl	3.5-5.2 g/dL
Total protein	7.0 g/dL	6.8 g/dl	7.2 g/dl	6.6-8.3 g/dL
Aspartate transaminase	28.9 U/L	34 U/L	32 U/L	2-50 U/L
Alanine transaminase	19.14 U/L	26 U/L	28 U/L	1-50 U/L
Coagulation profile				
Prothrombin time	12 seconds	13 seconds	13 seconds	10.7-14.3 seconds
International normalized ratio	1	1.1	1.1	0.8-1.2
Activated partial thromboplastin time	28 seconds	30 seconds	29 seconds	25-35 seconds
Occult stool blood test	Negative	Positive	Negative	Negative
*Helicobacter pylori* stool antigen	Negative	-	-	Negative
Serology				
Hepatitis B surface antigen	Negative	-	-	Negative
Hepatitis C virus antibody	Negative	-	-	Negative
Rapid HIV test	Negative	-	-	Negative

Intraoperatively, spinal anesthesia was administered, and the patient remained awake and communicative during the initial phase. Approximately five minutes after insertion of the PMMA bone cement, the patient developed sudden and profound cardiorespiratory compromise, characterized by a precipitous drop in oxygen saturation to 40%, severe hypotension with blood pressure measuring 50/30 mmHg, and marked bradycardia at 30 beats per minute. Despite the prompt initiation of resuscitative measures, including administration of supplemental oxygen, rapid intravenous fluid infusion, and intravenous hydrocortisone, her condition continued to deteriorate and progressed to cardiac arrest within minutes. The surgical site was packed, cardiopulmonary resuscitation (CPR) was initiated promptly, and the patient was intubated and ventilated with 100% oxygen. Intravenous adrenaline was administered, and return of spontaneous circulation was achieved after four cycles of high-quality CPR. The procedure was subsequently completed within one hour, with an estimated blood loss of 100 ml.

**Figure 2:**
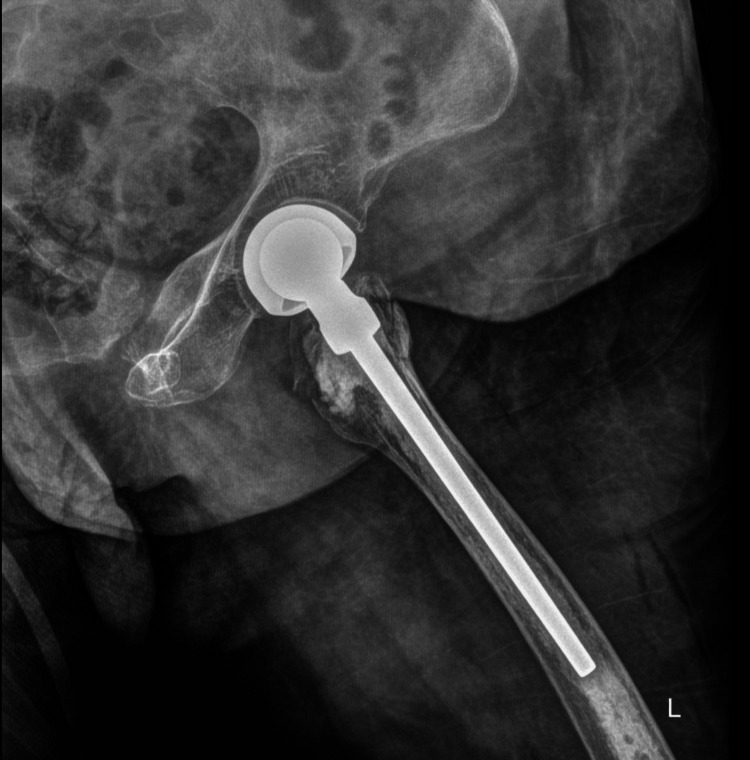
Postoperative radiograph showing proper alignment and positioning of the left hip implant

Postoperatively, the patient was admitted to the intensive care unit (ICU), where she was managed with mechanical ventilation and continuous infusions of norepinephrine and epinephrine. She received one unit of packed red blood cells, and over the subsequent 48 hours, vasopressors were gradually tapered; she was then successfully extubated. A chest CT angiography was performed to rule out pulmonary embolism and demonstrated no evidence of pulmonary embolism, parenchymal infiltrates, pleural effusion, pneumothorax, or other intrathoracic abnormalities. A postoperative radiograph of the left hip demonstrated correct implant alignment and positioning (Figure 2).

After one week in the ICU, the patient was transferred to the surgical ward in stable condition, where she was initiated on physiotherapy. Four days later, she was discharged on oral tramadol and paracetamol, along with subcutaneous enoxaparin. At her one-month postoperative follow-up, the patient exhibited marked functional improvement. She was ambulating with the assistance of a cane and reported no significant pain or complications. Clinical examination revealed a well-healed surgical wound, and there were no signs of infection or implant-related issues.

## Discussion

BCIS is a potentially fatal complication associated with the use of PMMA during cemented orthopedic procedures, most commonly hip hemiarthroplasty, but also reported in total hip and knee arthroplasty. Although no universally accepted definition exists, BCIS is recognized as a significant cause of intraoperative morbidity and mortality [1,4]. Clinically, it is characterized by sudden hypoxia, systemic hypotension, pulmonary hypertension, bradycardia, cardiac arrhythmias, loss of consciousness, and, in severe cases, cardiovascular collapse or cardiac arrest [1-3]. These manifestations most often occur intraoperatively at critical points, such as cementation, prosthesis insertion, or joint reduction; however, milder forms of BCIS have also been observed in the postoperative period [1].

Donaldson and colleagues proposed a grading system for BCIS based on blood pressure, degree of hypoxia, and level of consciousness, with higher grades corresponding to worse prognosis and the final grade requiring CPR. The BCIS grading system defines three levels: Grade 1, characterized by oxygen saturation (SpO₂) <94% or a decrease in arterial systolic blood pressure (SBP) of >20%; Grade 2, defined by SpO₂ <88%, SBP decrease >40% from baseline, or sudden loss of consciousness; and Grade 3, which involves cardiac arrest or respiratory failure requiring CPR. This classification is clinically valuable, as it guides monitoring and timely intervention, with higher grades indicating increased risk of morbidity and mortality [5]. In the present case, our patient developed severe intraoperative BCIS corresponding to Grade 3, necessitating advanced resuscitative measures.

The pathophysiology of BCIS is multifactorial and not fully elucidated. One principal mechanism is embolization: during cement pressurization, medullary contents such as fat, marrow, air, and particulate matter are forced into the venous circulation, leading to pulmonary embolism, increased pulmonary vascular resistance, and right ventricular failure [5]. Additionally, the exothermic polymerization of PMMA bone cement can cause local tissue damage, resulting in the release of histamine and prostaglandins, which may trigger a systemic inflammatory response that exacerbates respiratory and circulatory failure. Complement activation and methylmethacrylate monomer-mediated vasodilation have also been proposed as contributing factors. These mechanisms collectively contribute to the acute hemodynamic instability observed in BCIS [1,6,7].

Risk factors for BCIS include advanced age; impaired cardiopulmonary reserve, such as right ventricular dysfunction, coronary artery disease, or pulmonary hypertension; and skeletal conditions like osteoporosis, which may facilitate emboli formation during cementation. Additional factors associated with an increased risk include intertrochanteric or malignant fractures, metastatic bone disease, a wide or previously uninstrumented femoral canal, and the presence of a patent foramen ovale, which can facilitate paradoxical embolization [8,9]. In the present case, several of these factors were relevant. The patient was 90 years old and had long-standing hypertension and mild aortic regurgitation, all of which likely limited her physiological reserve. These vulnerabilities, combined with the stress of cementation, likely contributed to the rapid development of severe hypoxia, hypotension, bradycardia, and cardiac arrest observed intraoperatively. Several surgical strategies can help mitigate the risk of BCIS. While cement-free prostheses eliminate the risk, risk-reduction techniques include opting for a short-stem prosthesis, using low-viscosity cement, thoroughly lavaging the intramedullary canal, and ensuring hemostasis before implant placement [1,5].

Alternative diagnoses such as massive pulmonary embolism, acute myocardial infarction, and anaphylactic shock were considered but ruled out. The patient had normal cardiac troponin levels, and a post-event chest CT angiography showed no evidence of pulmonary embolism. There were no signs of anaphylaxis, such as cutaneous manifestations or bronchospasm, and no identifiable exposure to known allergens during the perioperative period.

Management of BCIS requires prompt recognition of early clinical manifestations, such as sudden desaturation, hypotension, bradycardia, or loss of consciousness, which should immediately trigger resuscitative measures. These include the administration of supplemental oxygen, intravenous fluids to maintain preload, and vasopressors to support blood pressure. In cases of cardiac arrest, advanced life support protocols should be initiated without delay. Postoperative care often involves intensive monitoring in a critical care setting, with support for respiratory and cardiovascular function as needed [10]. The prognosis can be favorable if interventions are timely and effective, as demonstrated in cases where patients have recovered with appropriate management.

This case highlights the importance of vigilance and preparedness in managing patients undergoing cemented hip arthroplasty, especially those with known risk factors for BCIS. It underscores the need for a multidisciplinary approach involving anesthesiologists, surgeons, and critical care teams to ensure prompt recognition and management of this potentially fatal complication.

## Conclusions

BCIS remains a rare but potentially catastrophic complication of cemented hip arthroplasty, with outcomes ranging from transient hypoxia to intraoperative cardiac arrest. This case highlights the importance of recognizing patient-related risk factors, maintaining vigilance during cementation, and ensuring prompt initiation of resuscitative measures when BCIS occurs. A practical intraoperative checklist for high-risk cemented hemiarthroplasty includes pre-cement pause, administration of 100% oxygen, alerting the anesthesia team, availability of vasopressors, medullary venting/lavage, gentle pressurization, and close post-cement hemodynamic monitoring. Multidisciplinary preparedness involving anesthesiologists, surgeons, and critical care teams is essential to improving patient survival. Awareness of the condition, meticulous perioperative monitoring, and early intervention can significantly influence prognosis in this high-risk population.
